# Obscure Trunk Pains

**Published:** 1861-07

**Authors:** 


### OBSCURE TRUNK PAINS.

OBSCURE TRUNK PAINS; OR, CHRONIC PAINS IN THE ABDOMINAL AND THORACIC WALLS. By *James Jago*, M.D., Oxon, Physician to the Cornwall Infirmary.—The great multitude of patients who seek advice on account of obstinate pains in the walls of the chest or of the abdomen, and the obscurity that frequently hangs over the origin of their sufferings, must always claim the indulgence of the physician for any attempt to throw another ray of light on their modes of production. I shall, therefore, venture a few remarks on the subject, premising that these will be of an elementary character, chiefly aiming to show that the weight of the upper portion of the frame often contributes to engender sufferings in those parts, in ways not commonly suspected; and that thus there may be simple means at hand of affording relief in cases beyond the reach of drugs. While such is my main purpose, I may be tempted to illustrate the views submitted from collateral sources.

It is an old remark* that rheumatism is oftener found associated with the tendinous and aponeurotic continuations of muscles, especially at the junction of these with bone, than with the muscular fibre itself. And Dr. Inman, of Liverpool, has ably maintained† that much of the pains experienced in the structures now mentioned are due to a stretching of fibrous tissue by the contraction

---

* Copland's Medical Dictionary, vol. iii. pp. 616–9.

† Spinal Irritation, or Myalgia, 8vo., 1858; second edition, 1860.

of muscle, or by its excessive tension produced by any other means. He elaborately distinguishes between the pains thus engendered in the muscular fibre, as cramp or spasm, and those resulting from strain along the line of osseous attachment, or line of union between the muscular and tendinous fibres. And he insists that in a weakened condition of the animal system the muscles are more liable to cramp than in a robust, and pain more likely to be felt along the said lines of junction.

Now, I have no further exception to take against this doctrine, but that I do not think that it embraces the whole series of such kind of pains as it aims at accounting for. I would affirm rather, in general language, that all the tissues of the body in their several degrees become painful when they are either stretched or *compressed;* in other words, that there is a state of equilibrium for the structures throughout the animal frame, which is the only one in which they remain free from pain: that the amount of suffering consequent upon the disturbance of this balance of opposing forces depends ordinarily upon the extent of the displacement, or the time during which it is kept up, that is, making allowance for the higher nervous endowment of one part than another, and taking for granted that in such a case the structural points to which the nerves of common sensation or their ultimate subdivisions are distributed, alone to be squeezed or stretched; for if a nervous trunk be thus affected, painful phenomena will, of course, accrue, radiating beyond the point mechanically interfered with.

To explain what I have just stated by an example. In a position of ease, the hand rests pretty much flexed. But, if we spread it flat upon a table, we soon begin to feel inconvenience from the tension of its skin and other tissues. And if we close the fingers tightly upon the palm, we soon find their tops in the fist becoming sore.

Or I may take another example, which may be regarded as furnishing an epitome of the principles I wish to apply in this paper. The eyeball is a globe suspended, as it were, by its muscles, in a fatty bed, and rotated so gently and equably as to preserve usually its proper form. If we make the slightest pressure of the finger through the eyelid over the sclerotic coat, we become informed of the compression of the retina along the line of greatest flexure by a luminous image of the contour of the applied portion of the finger; while we feel pain along the corresponding portion of the ocular globe—the visual sentient elements in which the optic nerve ends, manifesting in this way the effects we experience in the tactile sentients. In like manner if we strain the eye by resolutely staring in a given direction, the orbital muscles warp the globe out of form, false impressions of light result, and the power of seeing external objects is almost obliterated, while the orbital muscles are thrown into spasm, insomuch that we have a feeling of soreness and fatigue in them, with a tenderness of the ball itself, for many an hour after the experiment. Even any lengthened exertion of the eyes in our habitual occupations is attended with such results in an appreciable manner.

These illustrations will, I hope, help to a readier apprehension of our peculiar liability to sufferings of this order in the regions of the thoracic and abdominal walls, if it shall be made to appear that these are very variously exposed to be forced out of the position of structural balance, and that force applied hereabouts in one part often entails a displacement of the greatest magnitude in some others, in a manner that is not usually thought of.

Let us imagine, as a rough approximation, all the human body, exclusive of the lower extremities, in the sitting posture, as a heavy lever or a beam revolving about the *tubera ischiorum*, as an axis or fulcrum. And let us assume that the centre of gravity of this beam lies between the middle of the *scapulœ*. Then in the upright sitting position the whole weight of the beam falls upon the *tubera*. In the horizontal the weight will be borne up—distributed—along the whole length from the head to the buttock. If we recline, so that the back rests at some point against a prop, we may find the portion of the weight supported by the prop, by drawing a perpendicular to the beam at the point of contact with the prop, and then a perpendicular to this line from the fulcrum, as also a perpendicular from the fulcum to a vertical line passing through the centre of gravity: when the pressure upon the prop will be to the whole weight of the beam, as the latter of the perpendiculars let fall from the fulcrum to the former. Thus, the nearer to the buttock the prop is placed, and the more the body is made to recline, the greater pressure falls upon the prop. While it is apparent that should we, while we sit, lean pretty much back against a single prop meeting the spinal column at any point whatever, a considerable pressure must be endured by that part of the body in contact with the prop, which is a general statement sufficiently accurate for the purpose of a practical essay like the present.

Now let us further assume that the back while we sit is supported entirely by a *couple* of props of equal height, and several inches asunder, bearing each, similarly, against one of a pair of ribs. In this instance pain from compression would be felt at the points of contact, as is obvious. But there result also certain secondary effects which, I think, we are apt to overlook. The two ribs are made to revolve, by the pressure, upon their vertebral articulations, and the numerous ligaments that bind them to the vertebræ and neighboring ribs are stretched, and pain is occasioned in those parts. In addition to which, this movement causes an alteration in the shape of the ring made—with the interventions of the vertebral column, their cartilages, and the sternum—by the pair of ribs. Had such a ring as this been of uniform texture and shape all round, one effect of letting it bear merely its own weight upon two of its under points, in the manner here imagined, would be that of flattening the ring above. In such a combination of structures as do exist in these rings, the result must somewhat differ. Yet we may be sure of this much, that in the case before us much displacement must occur along the most pliant part of the ring—that is, along the cartilaginous portion, and at the articulations of the cartilages with the sternum: for the costal rotation at the spine tends to bring the sternal ends of the ribs nearer to each other, that is, to bend them at their junction with the sternum, independent of the weight of the upper portion of the ring depressing that region. In other words, pain would be developed at other parts than those where the direct pressure is applied.

Had we placed the two props much farther apart, and so as to take the weight of the trunk at the back part of a lower rib on one side, and of an upper one on the other, consequences very similar to those detailed in the case of a *pair* of ribs must have ensued. The ribs would have been pressed in upon the lungs behind, the cartilages would have been bent, and the sternum itself—in young subjects at least—somewhere along the imaginary line drawn diagonally across it, that joins the articulations with the sternum of the two ribs selected. This may be regarded as a general sketch of what takes place in such an experiment; but it must not be forgotton that a pressure received by any rib will in some degree be transmitted to adjacent ones. And it can scarce need to be added that if our back be supported at more than two points, or even along its whole length, the effects here described will only be diminished in amount, and nòt be entirely obviated.

Now, in the sitting posture, if the support of the back happen to be a flat surface, as in a church pew or box in a theatre, the back rests against the shoulder-blades, that is, virtually the upper ribs; and, *a fortiori*, if the support for the back consists, as in most chairs, of a series of a few horizontal concave bars, the ribs are likely to have to bear, at a distance from their vertebral articulations, a large amount of pressure. In weakly persons such a posture produces—not to dwell on that occurring posteriorly—suffering in the *anterior* part of the chest, especially along the margin of the sternum. For not only from what has been stated must this happen, but, in addition, the thrusting forward of the shoulders renders the pectoral and great serrate muscles lax, and thus deprives the ribs of the support due to the elasticity of these inspiratory muscles, increasing the liability to the kind of inconvenience we are discussing; while a confined play of the respiratory movements is induced, entailing other evils. Even lying upon the back in bed—more completely still so if on a hard mattress without elasticity enough to expand itself decidedly against the spine along the interscapular gutter—will leave the scapulæ—that is, the upper ribs—to bear a weight at least equal to that of the whole thorax, and will occasion a strain along either margin of the sternal gutter.

Having considered some effects upon the thoracic walls of flexure in a plane perpendicular to the spine, let us now turn our attention awhile to flexure in planes passing through its axis.

We will take first the case of a person sitting on a chair whose back does not rise to his shoulder-blades. As he reclines, the centre of gravity of the beam falls above the point of support, and the beam is only prevented from lifting at the bottom by the weight of the lower extremities. Indeed, did we propose to break a stick over the back of the chair, we should put it under the same conditions, confine the end of the shorter branch, and apply our strength (the weight of the head and shoulders in the example of the beam) at the end of the longer one. We see the stick split on the side opposite to that which rests on the fulcrum, because the forces applied tend to lengthen that side, and strain most those fibres which are most remote from the fulcrum. Just so the whole line of tissues along the paths of the recti abdominis muscles and the sternum, from the pubis to the clavicles, are laid under extreme tension; the ensiform cartilage and the whole sternum is depressed toward the spine, and the pleural cartilages warped. And pain again befalls the region.

It may be appended that the actual *fixed* point at which the trunk curves backward is not where the spines of the vertebræ are in contact with the back of the chair, but rather at the posterior margins of the bodies of the said vertebræ, and that the spines gather nearer together and the skin of the back into transverse folds, as we may have observed the skin covering the stick, being broken as above, to do on the side next the fulcrum, because the fixed point, or the unstretched longitudinal fibre, is at the back of the stick itself. Again, if the sole transverse portion of the chair-back consist of one bar at the height, say of the root of the sitter's neck, unless great muscular effort be made, the weight of the loins and thorax would cause the trunk to become convex behind, just as occurs when we stoop to pick anything from the ground; and we know how liable such a stretching of the lumbar muscles is to be followed by crick, and that it always takes a good interval to recover from the sorenesss resulting from a prolonged stooping posture. But as the line that undergoes, in this instance, no change in length, is somewhere along the anterior edges of the vertebræ, the lower end of the sternum is brought much nearer than before to the pubis, the contents of the abdomen pressed up toward the cavity of the chest, and the abdominal muscles project forward and cover the ensiform cartilage, so that this, by their support, takes some of the weight of the head and neck, and may thus be flexed, depressed toward the spine, carrying with it the lower costal cartilages of the left side, rather than those of the right, which are supported by the liver. So that this very pain may be produced in the district so much exposed to this misfortune.

If we press upon the sternum directly, as in leaning over a table to write, we may push it toward the spine and bend the attached cartilages. If we curve the trunk laterally, as in leaning on a table sideways, we stretch the opposite oblique muscles, and may occasion pain either at their upper costal insertions or at their lower pelvic. The same effect may follow by lying in a bed; for on the side on which we lie, by the weight of the lumbar portion of the trunk, the natural concavity of the waist tends to become obliterated; for this portion of the body is now primarily supported at the brim of the pelvis and lower true ribs.

I must here conclude my anatomy of trunk-pains, as I believe I have indicated the fundamental way in which the commonest of the chronic kind are generated. It were an indefinite task to work at exhausting the subject. The weight of the arm may make the muscles of the shoulder ache; and it is conceivable that one might write a separate history of the sufferings of all the muscles of the trunk. Besides, the effect of the compression or weight of clothes might be expatiated upon. Such effects as these, I presume, are likely to be detected with little difficulty, by those familiarized with scrutinizing the sources of pains in the thoracic and abdominal walls, by first eliminating those that may arise in any of the modes above described. As far as I have here gone I am persuaded that I have not transgressed the bounds of clinical experience, and that there is not one of the ways of suffering just pointed out which I have not met with in several instances, and seen relieved simply by precautions against the strain that occasioned it.

To give cases in detail would only be repeating in the form of narratives the foregoing explanations, so I will content myself with a simple sketch as a type of the class. I once had to think for a young gentleman of rather studious habits, of a slender, symmetrical figure, but enjoying good health, except that he was rendered miserable by attacks of pain at the sternum, and along the cartiages of the ribs, commonly about the lower end of the sternum, though sometimes higher up—a condition of things that existed for two or three years in spite of tonic and other medical treatment. But he having himself observed that he had once been extremely afflicted after pressing the epigastrium against a table in drawing, it soon came to light that all his sufferings arose from posture. Even lying upon his back in bed was found to bring on a fit of pain, and resting his back against any support that threw the weight rather upon the ribs than the spinal column did the same. He now soon got rid of all his troubles by habitually supporting his back, when studying, at the spine. I omit to state how, as I mean to consider the best mode of doing this in another part of this paper.

Perhaps it may not be superfluous to subjoin, that such a relative depression of a rib with respect to those next it, as may happen when it has to bear undue pressure in some of the modes above treated of, may sometimes expose the trunk of an intercostal nerve to squeezing or stretching. These nerves are, indeed, curiously lodged in grooves along the lower borders of the ribs, which guard them from accident; but they traverse a short space from the spine to enter these, and do not seem to lie absolutely safe from mechanical injury. I am not sure that I have not met with cases of neuralgia shooting along their paths from this cause, wherein no spinal deformity existed. It is the nerves most unprotected against pressure and cold that appear most liable to neuralgia. I am confident I have known sciatica to be engendered by a habit of sitting sideways, in a partially recumbent manner, on a sofa or chair, so as to squeeze the sciatic nerve at its emergence from the pelvis into the thigh; and several instances in which a fit of tic douloureux has followed a nap upon a book or some hard thing for a pillow. Nevertheless, these nerves cited in illustration are oftener affected by their exposure to great differences of temperature, since even the sciatic, by careless sitting upon surfaces which rapidly conduct heat, are subjected to such vicissitudes.

In juxtaposition with the reflections with which this paper sets out, though I would be chary of applying them too far, I have reason for surmising that there are some ailments whose seats are among the abdominal or thoracic viscera themselves, instead of in the trunk walls, which can only be understood by such rudimentary considerations. I will touch upon two or three rarer examples which have fallen under my own notice.

Awhile since a woman of about thirty-six years of age became a dispensary patient of mine, for what, *prima facie*, seemed to be a form of gastralgia common enough among such patients. She asseverated that she literally feared to eat a full meal, so greatly did she suffer pain at the pit of the stomach, which radiated along the lower border of the thorax for an hour or more after a meal; that she had become very thin in consequence of enforced abstinence, and that several medical men had failed to give her any relief. Though her affirmed loss of flesh seemed justified by her appearance, and her look of distress was great, yet she had not the aspect nor any of the prominent symptoms of organic disease of the stomach; there was no chain of evidence to convict it of scirrhus or ulcer. It was not until I had exhausted in vain all the current remedies for facilitating digestion that, upon a more deliberate sifting of the symptoms, I ascertained the curious fact that if she went to bed immediately after eating supper, this meal gave her but little inconvenience. Thereupon I directed her to lie down after all her meals, and to eat good solid dinners of animal food and vegetables, drinking bottled porter with them. With some difficulty I got her to persevere in this practice, and she was not many weeks before she was convalescent. Now, we cannot imagine that mere distention of the stomach occasioned the suffering, because the recumbent posture could not obviate such an effect; nor that there was an abrasion of the mucous membrane so placed in the organ as to be brought more into contact with the food in one posture of the body than another, for whatever may have been fancied to the contrary, the pressure of the atmosphere must keep the stomach always closed tightly upon its contents. It seems to me that the pain was evolved through the *weight* of the meal—by traction upon the diaphragm at the cardiac orifice possibly, but mainly by traction upon the liver by the lesser omentum, and thereby upon the xiphoid and costal cartilages uniting with it. I would compare the case with the two following.

About the same time, I had under my care two cases parallel to each other in all essential particulars. The first was that of a miner who had been for more than half a year under treatment for what he described as a most severe pain along the margin of the cartilages of the lower ribs on the right side, extending from the edge of the liver downward, through the abdominal wall, and pentrating, he fancied, inward. He shrunk when I touched the abdomen over the parts referred to. It was only with difficulty he could stand upright, or walk, or rather crawl, about. Being unable to discover anything amiss with the liver, or indeed, at first view, any general sign of ill health, except lack of flesh, I commenced a devious examination of his body, and, to my surprise, found that he had effusion into the pleura, not on the side complained of, but on the left. Posteriorly and laterally the bottom of the chest on this side was much duller on percussion than on the corresponding portion of the right side, notwithstanding the hepatic dullness here existing. At the same time respiration on the left was barely audible at levels, where on the right it was very so; and fremitus on the left was almost as barely perceptible, while the intercostal spaces protruded. By putting him under a succession of blisters over the diseased region, instead of, as had before been done, over the region of pain, with iodide of potassium, diuretics, and then tonics administered internally, the physical signs of effusion soon began to diminish, and with this the pains in his right side.

A few months afterward a once stout and lusty smith, who had been working in London, returned to Cornwall in a very weak state, wasting and laboring under excessive pain at the right hypochondrium, and spreading over the abdomen, which pain he accused as the sole cause of his troubles. He had been perfectly well until about six months before, when, after an imprudent exposure to a piercing cold draught of air, he became suddenly ill, his suffering settling soon entirely in his right side. This man's story and attitude reminded me so forcibly of the foregoing case that I at once examined his chest, and discovered more strongly marked signs of pleuritic effusion on the left side than in that of the miner—a condition of things which, as in that instance, had been overlooked. He commenced, too, to recover from the moment the true seat of disease was besieged.

With respect to the propagation of the pain to the opposite side in this pair of cases, it seems to me that the mode might have been thus: The fluid in the left pleural sac would have nothing to sustain its weight beneath the diaphragm but yielding viscera, consequently the left half of that muscle would be depressed below its usual region; but this could not happen without dragging downward the right half also—that is, not without pulling downward upon the liver, and exerting a force to rotate it on its anterior border from the costal cartilages which cover it. In this way not only would these cartilages themselves be warped, but the ligaments of the liver would be made abnormally tense, and therefore their peritoneal continuations along the parietal abdominal wall be unduly stretched, as well as such tissues as it lines. I venture on this conjecture on taking *common* survey of the three cases last introduced, under guidance of the more general principles on which this paper proceeds.

*Infra-mammary Pain.*—I must emphatically repudiate the idea that I would offer the preceding propositions as a key for opening the mysteries of *all* the aches we meet with in the thorax and adjacent parts. In such an affection as hysteria, for example, perturbations of all the other parts of the nervous system are far too transparent for me to go so far as to say that the tactile nerves, or such nerves, if they be other than these, whose office is to inform of violence done to the tissues, may not also be morbidly affected. Nevertheless, let us see whether the pain, under the classic designation heading this paragraph, admits of a plausible explanation by the principles submitted. In the autumn of 1858 this pain was made a topic of discussion in the medical weekly publications. Dr. Inman, Dr. Fuller, Mr. Holmes Coote, and others took part in the debate. I do not know that I can do better than take the main points at issue from their statements.* The first of these writers, in reply to the second, who had asked him why the seat of a chronic pain is more commonly in the left than in the right side, remarks: "At one period I thought I could trace some connection between the locality of the pain and the position commonly adopted by the sufferer; but after a more extended inquiry I have been obliged to consider the facts referred to as inexplicable in the present state of our knowledge."†

Mr. Coote‡ comments upon this correspondence. Premising that it is not to be doubted that it may have "more causes than one," he affirms that he has noticed it to be "one of the very earliest and commonest symptoms of incipient lateral curvature of the spine," and he reminds us that it had been recognized in this point of view since the days of Delpech, (citing his "Orthomorphie," tome ii. p. 10, 1828;) "a constant pain, somewhat vague in its seat, which takes place sometimes in the side of the chest below the mamma, sometimes in the epigastric region. This pain has no known cause; its duration is usually constant, but it is infinitely variable; its periods of calm and exacerbation have nothing regular; there is no disturbance of the functions of the organs in the seat of pain; nothing quiets it, nothing relieves it. It is accompanied by slow, progressive, and inexplicable deterioration of the state of health. It is evidently allied to something grave but quite clandestine." Delpech then instances a girl of eleven years thus afflicted, who, by means of a plumb-line, was found to

---

* I have not access to the *second* edition of Dr. Inman's "Myalgia;" I am therefore unaware if he has therein made any new observations upon this subject.

† British Medical Journal, November 27th. ‡ Ibid., December 4th.

have a slight lateral curvature. "The greater frequency of spinal curvature," Mr. Coote subjoins, "may explain in some measure the obedience of infra-mammary pain to the same law." While thus suggesting that it may not improbably be muscular, he does not indicate by what mechanism the curvature or the pain inclines to one side rather than the other.

However, as Mr. Coote had (as recently as in the *Lancet*, October twenty-third of the same year) given a graphic sketch of the course usually taken by spinal contortion, I will continue to follow him: "The curvature generally commences in the upper dorsal region, and extends directly to the right in one even sweep up to the junction of the lower dorsal with the lumbar vertebræ; then the direction of the articulating surfaces is altered, and the movements of the spine change from the lateral inclination to the antero-posterior movement, as in springing; a second curve forms in the lower region to the left, accompanied with a rotation and twisting of the vertebræ." He then proceeds to quote from the plates and descriptions of M. Bouvier, (Physician to the Children's Hospital, Paris,) explanatory of the packing of the viscera found on dissection of cases of confirmed lateral curvature of the spine. Thus, in what he calls *le premier plan*, that of the above type, the latter says the "lungs are apparently not much deformed, when viewed anteriorly. But the right is reduced in height, its base being pressed by the abdominal viscera; the left has its inferior lobe compressed between the ribs and the heart. The heart is voluminous, and closer than natural to the ribs of the left side. The liver is much deformed, deeply fissured, and, as it were, mounted (*à cheval*) on the crest of the ilium."

Now, it is worthy of consideration whether we may, by chance, find the cause of the preference of the line of lateral curvature for one side of the body rather than the other, in a difference in the compressibility of the viscera that are lodged against the parietes of the respective sides. If there be any just foundation in this notion, the only organ within that seems at all capable of affording a relatively greater support is the liver. This is a firm, resisting mass, filling the whole of the right hypochondrium; and if the spine becomes drooping and weak, or its muscles no longer able to sustain the upper portion of the body erect; if the vertebral column begins to yield, so that a weight that it should carry becomes cast upon the viscera, it seems not absurd to regard it as not improbable that the circumstance of the left lower ribs and cartilages covering compressible lung and stomach, while the right are supported all along within by a much firmer body, may be the means of causing the chest to arch inward on the left rather than the right.

The details of M. Bouvier give strong countenance to the idea that the left pleural cartilages are more liable than the right to be warped out of shape by any of the modes suggested in the beginning of this paper. And thus the left side will be more frequently the seat of pain than the other. By whatever cause of ill health the general strength fails, this liability will be more remarked. This seems a necessary consequence of the manner of stowing the more massive viscera in the chest and belly. We may assert this much without pushing our conclusion so far as to assume that infra-mammary pains can be of no other order. I can well conceive that the site of the liver may determine that we are not left but *right* handed, because the pectoral and other muscles that move the arm or shoulder upon the trunk have, through the hepatic support, steadier points of resistance on this side than the other; for this organ manifestly plays an important mechanical as well as a more purely glandular part in the economy.

Having so nearly approached the subject, perhaps I may be permitted to say a word or two on the thoracic distortions in rickets. Dr. Jenner* speaks thus: "The deformity of the greatest interest to the physician is that of the thorax. The back is flattened. The ribs are bent at an acute angle where the dorsal and lateral regions unite. At that part the lateral diameter of the thorax is the greatest. From it the ribs pass forward and inward to the point where they unite with the cartilage; on that line the lateral diameter of the thorax is the least, the cartilages curving outward before turning in to unite themselves to the sternum. The sternum is thrown forward, and the antero-posterior diameter of the thorax is abnormally great."

"The great determining cause," he afterward subjoins, "of the thoracic deformity is the atmospheric pressure; this is aided by the elasticity of the lung," and the fact that the more resistant viscera underneath are obstacles to recession of the chest-walls where they lie. It appears to me difficult to account for the "acute angle where the dorsal and lateral regions unite," or the flattened back, by such diffusive pressure as must be produced between the air without and the air-containing elastic lung within. On the other hand, it is certain that if the rickety child be let to lie much on its back, which, from its inability to stand and sit upright, would inevitably happen, the weight of the chest must so press upon the softened ribs as to tend to bring the spines of the vertebræ and those of the shoulder-blades on the same level, or to flatten the back. Again, the mere weight of the costal cage of bone and cartilage tends to flatten it in front, (above,) so that this cage would tend to fall flat in front and to crack sharp off at the sides. Now, when we take into consideration with these facts the obstacle to recession of the cage in front furnished by such solid parts as the liver, heart, and spleen, as Dr. Jenner himself does, I think it may be worthy of reflection whether this is not the simple history of the development of the deformity in question. Allowing that some deviations must be expected to be produced in the form of the chest by weights thrown upon it when other postures are assumed, the principle involved in this explanation would be in accordance with Dr. Jenner's general mode of accounting for rickety distortions in the limbs, which he ascribes to their weight or the weight of other parts bearing upon them. He remarks, "in excluding muscular action from all direct share in the production of curvature of the long bones in rickets, I am, so far as I know, unsupported by any authority;" this observation, it may be implied, he would extend to the chest, as he cites Rokitansky's opinion of the thoracic deformity being caused by a want of power in the inspiratory muscles, and moots no other hypothesis but his own.

Finally, returning from these collateral meditations, I will venture a word as to the practical use of such considerations as form the fundamental reasonings of this paper. If suffering is occasioned in a patient by a disturbance of structural balance in any part, the obvious indication of cure is to remove the cause of the disturbance. If any body by pressing upon the eyeball deprives it of its

---

* Medical Times and Gazette, March 17th, 1860.

sphericity, remove the body that does so. If a patient leans against his sternum in writing till he begets suffering thereabout, take care he ceases to do so. Each case requires its special precaution so clearly that it were idle to iterate the fact. But I hardly think it unprofitable to invite attention to a very homely topic before I take leave of this essay.

It has been shown how important it is that the structural balance of the trunk should be ordinarily preserved. When we walk, this equilibrium is stable, and when we lie it is approximately just. But we spend a great portion of our time in the sitting posture, and what provisions have we that the balance shall be easily kept in this attitude? In plain English, on what principles are the backs and other upright supports of our chairs and sofas constructed? Our easy chairs and couches, not to say chairs for general use, show no conformity in the principles upon which those uprights are conceived. All looks as if there were no knowledge of comfort in such things. The majority seem made with a view to torture rather than ease. I cannot therefore deem it a thankless task to endeavor to ascertain what are the requisites of such structures. At least, I will give a device for what I have concluded to be the best form of chair as an example. I will do so in dimensions to fit the use of a man of middling size. The chair shall be of wood, for if it be easy in bare wood it cannot fail to be so when cushioned.

Let the seat be 18 inches from the floor and of the shape of a semicircle of a diameter of 21 inches, whose straight edge is the front of the seat, and let it, in a style which is common, be scooped toward the back to fit the nates. Again, in the usual style of elbow chairs, let there be a horizontal semicircular bar for the elbows to rest upon, 11 inches above the seat and of its diameter, and let this elbow-bar be supported on either hand laterally by four or five upright bars with equal intervals.

In the middle of the back let the support for the elbow-arch be a flat plank of 5½ inches' breadth, or rather, let two short elbow-bars be let into such a plank rising from the middle of the posterior border of the seat, to the height of 22 inches, and let this plank be curved to the lateral contour of the loins, as may be seen in the cushions of railway carriages. Or, in definite measurements, let the plank, at vertical heights of 2, 5, 10, 15, 22 inches, be gradually inclined backward to the horizontal extent of $\frac{1}{4}$, $\frac{3}{4}$, $2\frac{1}{2}$, $3\frac{1}{4}$, 4 inches respectively. Beginning at the top of the plank, let a longitudinal groove be cut a foot down the anterior face of the plank, of 1 inch wide and $\frac{3}{4}$ inch deep; let its edges be beveled away, as also other sharp edges of the plank. Below the elbow-arch the plank may be hollowed in the horizontal direction to coincide in curvature with the seat.

Graceful appearance apart, we have here the elements of a comfortable chair. The lower portion of the back will form a cradle for the pelvis, and prevent its weight from effecting pressure upon higher parts of the trunk; the curved portion rising therefrom will bear up the loins and lower part of the chest. The backpiece will allow the middle channel of the back to rest against it at any and every point, while its longitudinal groove will afford on escape from pressure for the thinly-covered vertebral spines, leaving this to be encountered by the bed of muscle at their sides. When any pressure that might be extended to the ribs would not affect the form of the chest, owing to the short leverage at the vertebral articulations of the ribs along the channels at the sides of the spine. The shoulders play backward and forward without impediment; and since the trunk preserves its natural form as in a standing position, the respiratory muscles meet with no hinderment in their office. The arms, resting upon the elbow-bars, do not cast their weight on the shoulders, while these are steadied.

But the combination of contrivances fulfills more recondite conditions. Not only do the ribs and abdominal surface ebb and flow as we breathe, but the curvature of the spine oscillates in degree. In this oscillation the backpiece allows the vertebral column to *roll*, as it were, along it—it is a sort of involute for the chain of vertebræ, as the evolute, to wind and unwind upon. For these reasons I have placed persons suffering from pains about the sternum, which have been acquired by pressure habitually thrown upon the shoulders, to sit against a narrow pillar or vertical plank, as may be met with in paneling, and the ready expedient has answered my expectations.

People do not sit long in one posture, and this chair allows a considerable degree of shuffling about without the sacrifice of its presumed advantages. If to change the point of chief pressure the nates be slid forward, the backpiece will still take spine, and chiefly at another spot, so as to relieve the points of pressure along the back also; and the semicircular shape of the back-cradle will give the sitter a fitting support for his back, however he leans about.

Though it has been laid down as a rule that the shoulders should not rest against any framework, it would not be amiss if there were such a framework forming a portion of a larger circle than the framework described, and crossing the upright bar at the top posteriorly, so that this bar be let into the upmost crosspiece in front, and that it project for an inch beyond it. Thus this extra framework, which may be partially formed by prolongations of the hinder upright bars that support the elbow-arch, would form recesses where the shoulders may be brought to bear a little to steady them laterally, and that they may take a little of the pressure of the trunk at our pleasure.

I have given the above description in stiff lines for the sake of being explicit and intelligible, but there is no reason why elegance should not be realized in the design. The elbows may be curved along their upper margin, the bars replaced by ornamental carving or wicker-work, the backpiece extended in a curved fashion below, or perforated for ornament, and may be undulating upward, spreading over the top of the shoulders on either hand. Cushioned chairs may be so stuffed as to afford a backpiece as here indicated, as well as the transverse projection for the loins as they now commonly have; and if carried as high as the head, bear a cushion projecting well *forward*, to take it, without the neck being unnaturally thrown back. I have thus devoted some attention to the analysis of the conditions essential to comfort in an article in daily use by every healthy person who is not destitute or a bed-lier; and whatever be thought of these reflections, it will not be gainsaid that they treat of questions of concern to the healthy, and of vast interest to a host of invalids.—(*British and Foreign Medico-Chirurgical Review*, April, 1861.)

